# Tibial Nerve Block: Supramalleolar or Retromalleolar Approach? A Randomized Trial in 110 Participants

**DOI:** 10.3390/ijerph17113860

**Published:** 2020-05-29

**Authors:** María Benimeli-Fenollar, José M. Montiel-Company, José M. Almerich-Silla, Rosa Cibrián, Cecili Macián-Romero

**Affiliations:** 1 Department of Nursing, University of Valencia, c/Jaume Roig s/n, 46010 Valencia, Spain; cecili.macian@uv.es; 2 Department of Stomatology, University of Valencia, c/Gascó Oliag, 1, 46010 Valencia, Spain; jose.maria.montiel@uv.es (J.M.M.-C.); jose.m.almerich@uv.es (J.M.A.-S.); 3 Department of Physiology, University of Valencia, c/Blasco Ibánez, 15, 46010 Valencia, Spain; rosa.m.cibrian@uv.es

**Keywords:** tibial nerve, supramalleolar approach, retromalleolar approach, injection site coordinates, success rate, regional anesthesia, ankle block

## Abstract

Of the five nerves that innervate the foot, the one in which anesthetic blocking presents the greatest difficulty is the tibial nerve. The aim of this clinical trial was to establish a protocol for two tibial nerve block anesthetic techniques to later compare the anesthetic efficiency of retromalleolar blocking and supramalleolar blocking in order to ascertain whether the supramalleolar approach achieved a higher effective blocking rate. A total of 110 tibial nerve blocks were performed. Location of the injection site was based on a prior ultrasound assessment of the tibial nerve. The block administered was 3 mL of 2% mepivacaine. The two anesthetic techniques under study provided very similar clinical results. The tibial nerve success rate was 81.8% for the retromalleolar technique and 78.2% for the supramalleolar technique. No significant differences in absolute latency time (*p* = 0.287), percentage of effective nerve blocks (*p* = 0.634), anesthetic block duration (*p* = 0.895), or pain level during puncture (*p* = 0.847) were found between the two techniques. The greater ease in locating the tibial nerve at the retromalleolar approach could suggest that this is the technique of choice for tibial nerve blocking, especially in the case of professionals new to the field. The supramalleolar technique could be worth considering for those more experienced professionals.

## 1. Introduction

Ankle blocking is a safe technique associated with a high success rate and a low risk of complications, and is very well accepted by both surgeons and patients [1]. Its main advantage compared with a combined femoral and sciatic nerve block is that there is no motor blocking above the ankle. This facilitates rapid mobilization of the patient, which is a relevant consideration in outpatient surgery, particularly in cases that require bilateral anesthetic procedures [2].

Of the five nerves that innervate the foot, the one in which anesthetic blocking presents the greatest difficulty is the tibial nerve. Several studies have agreed in locating the bifurcation of the tibial nerve at the tarsal tunnel level in a great number of cases [3,4,5,6]. However, a number of publications [3,7,8,9] note that the branching point of the calcaneal nerve shows considerable anatomical variation between individuals, which can contribute to a high rate of incomplete or failed blocks.

The particular interest in effective blocking of the tibial nerve is because a successful ankle block almost always depends on achieving satisfactory anesthetic blocking of this nerve. Moreover, in the majority of incomplete ankle block cases, the sensitivity is located in the region innervated by the tibial nerve [10,11,12].

The classic ankle block technique blocks the tibial nerve at the level of the upper edge of the medial malleolus [13,14,15]. The needle is placed behind the posterior tibial artery. However, the arterial pulse is not identified properly in many occasions. This considerably increases the difficulty for blocking tibial nerve, especially in the case of patients with edematous feet or peripheral vascular disorders. Furthermore, effective blocking of the tibial nerve necessitates blocking all three of its terminal branches: the calcaneal nerve, the medial plantar nerve, and the lateral plantar nerve. The first author to suggest a more proximal approach to the tibial nerve to ensure blocking of its three terminal branches was Gerbert in 1971 [16]. This author only suggested moving the injection site and gave no information on the success of the technique.

The objective of the present trial was to establish a protocol for two new tibial nerve block anesthetic techniques, to later compare the anesthetic efficiency of a retromalleolar tibial nerve block at the level of the most prominent point of the medial malleolus to that of a supramalleolar tibial nerve block injected 4 cm proximally from the lower edge of the medial malleolus, comparing as well the ease or difficulty of execution of each of the technique. Neither technique was based on the location of the posterior tibial artery pulse. The study hypothesis was that a supramalleolar tibial nerve block using a conventional technique could achieve a higher effective blocking rate, as the tibial nerve is less likely to have divided into its terminal branches.

## 2. Materials and Methods

### 2.1. Subjects

The study population was composed of surgical patients of the University of Valencia podiatric clinic (*n* = 133). In a single-blinded randomized controlled trial to ascertain and compare the anesthetic efficiency of supramalleolar and retromalleolar tibial nerve blocking, a total of 110 patients scheduled for foot surgery that required tibial nerve blocking were assigned at random to one of the two groups. Consort statement was followed for reporting parallel group randomized trials (see Table S1 in supplementary materials). The bases and conditions of this clinical trial were authorized by the Research in Humans Ethics Committee of the Experimental Research Ethics Commission of the University of Valencia (Spain) under procedure number H1477566491165. This clinical trial was registered with the ISRCTN registry (International Standard Randomised Controlled Trial Number) under number ISRCTN12176047. All the participants gave their written informed consent to participating voluntarily in the study.

To select the study sample, the following inclusion criteria were defined: subjects of both sexes, between 18 and 75 years of age, classified in the ASA Physical Status Classification System as ASA I (normal healthy patient) or ASA II (patient with a mild systemic disease that is well-controlled and with no functional limitations; for example, treated hyperthension), and requiring anesthetic blocking of the tibial nerve. Subjects with non-palpable peripheral pulses and/or an ankle edema that made it impossible to locate the anatomical reference points were excluded. Other exclusion criteria were coagulation disorders and/or infections at the target injection sites, a record of allergies to amide type local anesthetics, pregnancy or lactation, neurological or neuromuscular diseases, chronic analgesic treatment with opiate derivatives, and cognitive impairment.

A simple randomized list (allocation sequence) with numeric sequential unique identifiers was produced by software (Random Allocation Software 1.0 by Mahmood Saghaei from Isfahan University, Iran) for a sample size of 110 patients divided into two groups of equal sizes (allocation ratio 1:1). There were no restrictions such as blocking or block size. In this way, the participants were divided at random into two groups, which received either a supramalleolar tibial nerve block (SMB group, *n* = 55) or a retromalleolar tibial nerve block (RMB group, *n* = 55). The name of the group was placed in a sealed opaque envelope and the sequential numbers assigned to it were written on the front of the envelope. As the patients agreed to participate in the study, the auxiliary staff assigned them to the intervention groups, following the sequence.

### 2.2. Establishing Protocol of the Anesthetic Techniques under Study: Definition of the Injection Site Coordinates

**Figure 1:**
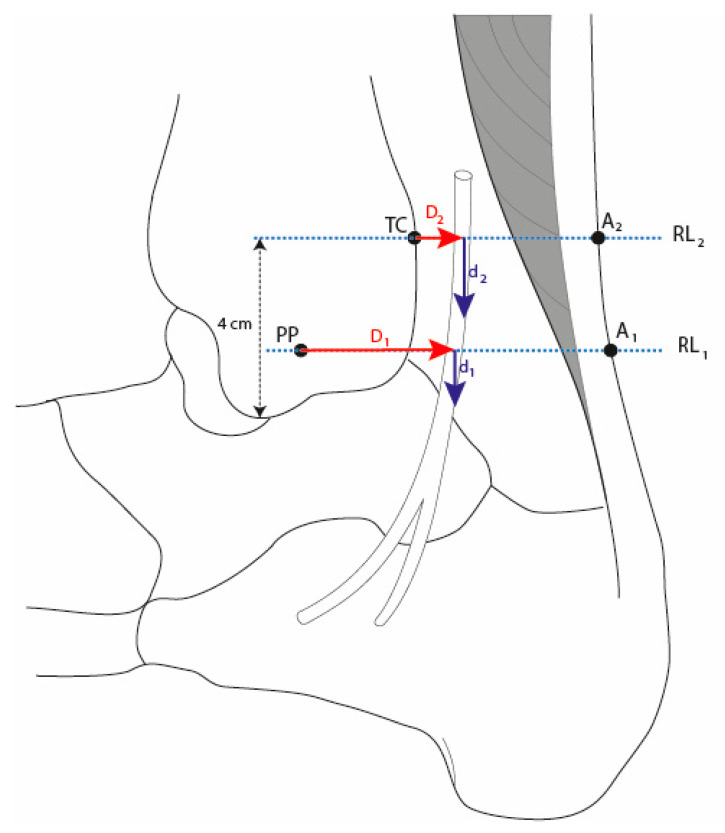
Diagram of the anatomical reference points for the retromalleolar and supramalleolar technique. PP = most prominent point of the medial malleolus, A_1_ = projection of a straight line from PP to the rear edge of the Achilles tendon, RL_1_ = retromalleolar reference line, D_1_ = distance from PP, TC = medial edge of the tibial cortex 4 cm proximally from the lower edge of the medial malleolus, A_2_ = projection of a straight line through TC to the rear edge of the Achilles tendon, RL_2_ = supramalleolar reference line, D_2_ = distance from TC, d_1_ = needle depth at retromalleolar approach, d_2_ = needle depth at supramalleolar approach.

**Figure 2:**
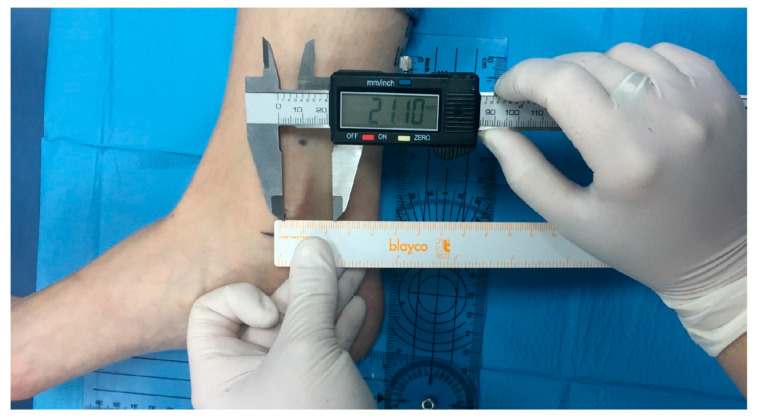
Retromalleolar injection distance coordinate. Calculation of the target injection site, based on the most prominent point of the medial malleolus, measured with a digital caliper.

The reference point for the retromalleolar technique was the most prominent point of the medial malleolus. The supramalleolar technique was performed 4 cm proximally from the lower edge of the medial malleolus (Figure 1). The injection coordinates (distance, needle depth, and needle angle) were obtained from prior ultrasound scans of the tibial nerve of 100 subjects in order to locate the anatomical position of the tibial nerve at the retromalleolar and supramalleolar levels based on the sex and the height of the subjects. These scans provided the anatomical coordinates for optimum identification of the injection site for tibial nerve blocking (Table 1). The distance coordinates for both techniques were calculated on straight lines between the respective bony reference points and the Achilles tendon, using a conventional 15 cm ruler. The exact injection site (distance from the reference point) was measured with a digital caliper with 0.1 mm accuracy (Figure 2). The needle insertion depth was marked with a sterile surgical marker. The angle of the needle was determined by the position of the tibial nerve within the vascular and nerve bundle (anterior, posterior, or lateral to the posterior tibial artery).

### 2.3. Experimental Procedure

For both techniques, anesthetic blocking of the tibial nerve was performed with the subject’s foot at a 90° angle to the tibia, using a goniometer for this purpose. A 23G 0.60 × 25 mL BL/L Braun^®^ needle and a conventional 5 mL syringe were used. The anesthetic solution administered was 3 mL of 2% mepivacaine without a vasoconstrictor (Scandinibsa^®^). Preanesthetic medication was not prescribed for any of the subjects.

**Figure 3:**
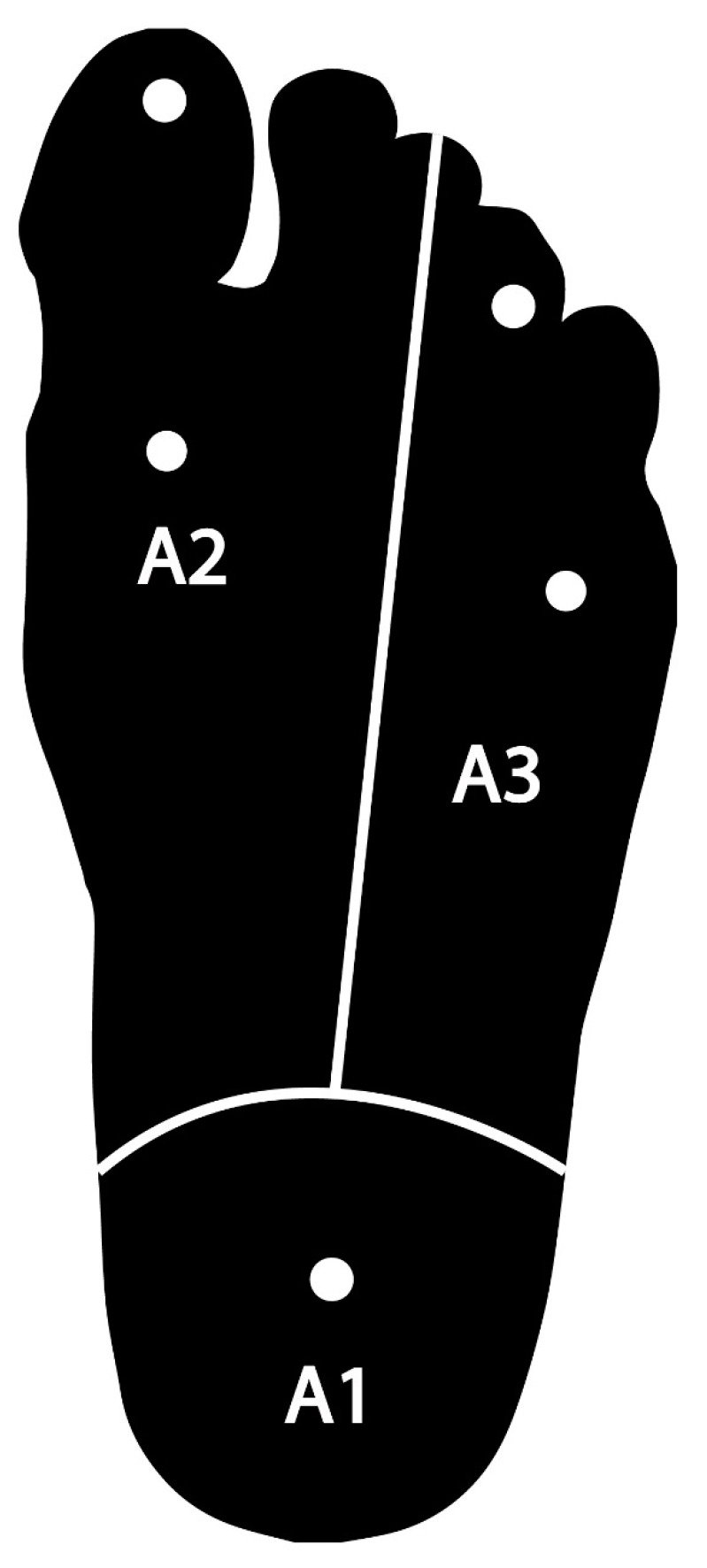
Division of the tibial nerve dermatome into three areas. A1 is the area innervated by the calcaneal nerve. A2 is the area innervated by the medial plantar nerve. A3 is the area innervated by the lateral plantar nerve. The circles mark the anatomical zones in which the two types of sensitivity (thermal and pain) were tested.

The primary outcome was the success rates achieved with the retromalleolar and supramalleolar techniques. This was determined by the anesthetic result (blocking effective, incomplete, or failed). The anesthetic result was measured by the extent of blocking, in other words, the number of areas with an absence of pain sensitivity and thermal sensitivity. For this purpose, the tibial nerve dermatome was divided into three areas (Figure 3). A1 was the region innervated by the calcaneal nerve, A2 was the region innervated by the medial plantar nerve, and A3 was the region innervated by the lateral plantar nerve. Absence of pain sensitivity was evaluated by pin-prick test with 21G needle. Absence of thermal sensitivity was evaluated by swab soaked in alcohol. Both sensitivity tests were conducted at five-minute intervals, starting five minutes after injection of the local anesthetic and ending on reaching the absolute latency time or 30 min after the blocking injection.

The tibial nerve blocking result, measured 30 min after initiating the block, was considered a failed block if the patient presented pain in the pin-prick test or sensitivity to cold in each of the three areas into which the tibial nerve dermatome was divided; an incomplete block if the patient only felt pain or cold in one or two of the areas; or an effective block if pain on pin-pricking and sensation of coldness were absent from all three areas.

In the incomplete or failed tibial nerve block cases, high-volume distal infiltration of a second tibial nerve block was administered prior to commencing surgery (7 mL of 2% mepivacaine, distal to the lower edge of the medial malleolus).

As secondary outcomes, the following were recorded: vascular puncture (positive aspiration), peri/intraneural puncture (electric shock sensations during puncture), the relative latency time (time in seconds from inserting the needle until the patient showed the first symptoms of anesthesia), the absolute latency time (time in seconds between inserting the needle and a complete absence of pain and thermal sensitivity in the sole of the foot), anesthetic blocking duration (time in hours between attaining blocking and the first appearance of paresthesias in the sole of the foot), the patient’s level of pain during puncture, and the need for intraoperative anesthetic reinforcement. The subject’s pain level during puncture was assessed on a 10-point visual analog scale (VAS) [17]. VAS scores of 0–3 were considered low pain, 4–6 moderate pain, and 7–10 intense pain. The need for intraoperative anesthetic reinforcement was measured by the number of patients who had attained effective blocking of the tibial nerve, but required additional local anesthetic during surgery.

All the anesthetic blocks were administered by the same person, the principal researcher in this study. The variables were analyzed and tested by an external observer who did not know which type of anesthetic block the subject had received and who was trained in evaluating and recording the findings as they were obtained.

### 2.4. Statistical Analysis

To calculate the sample size, the success rate of a conventional ankle blocking technique was assumed to be 66% in the reference population, as described recently by Chin et al. [18]. The present trial was expected to attain success rates of 66% for the retromalleolar technique and 90% for the supramalleolar technique. On this assumption, the necessary sample sizes were estimated as 44 patients in each of the two groups, accepting an alpha risk of 0.05 and a beta risk of 0.2 in a bilateral contrast. The expected loss to follow-up was 0%.

The data obtained were analyzed with IBM SPSSv22 software (Chicago, IL, USA). Means and the 95% confidence intervals (95% CI) were determined for the quantitative variables and percentages for the categorical variables. Student’s *t*-test was used to compare means. The equality of variances for the choice of the *p*-value of the Student’s *t*-test was valued by the Levene test. The Chi-squared test was used to compare percentages. A Kaplan–Meier survival analysis was performed with the log rank (Mantel–Cox), Breslow, and Tarone–Ware tests to explore the variables expressed as events over time. For all these tests, the significance level was set at *p* < 0.05.

## 3. Results

**Figure 4:**
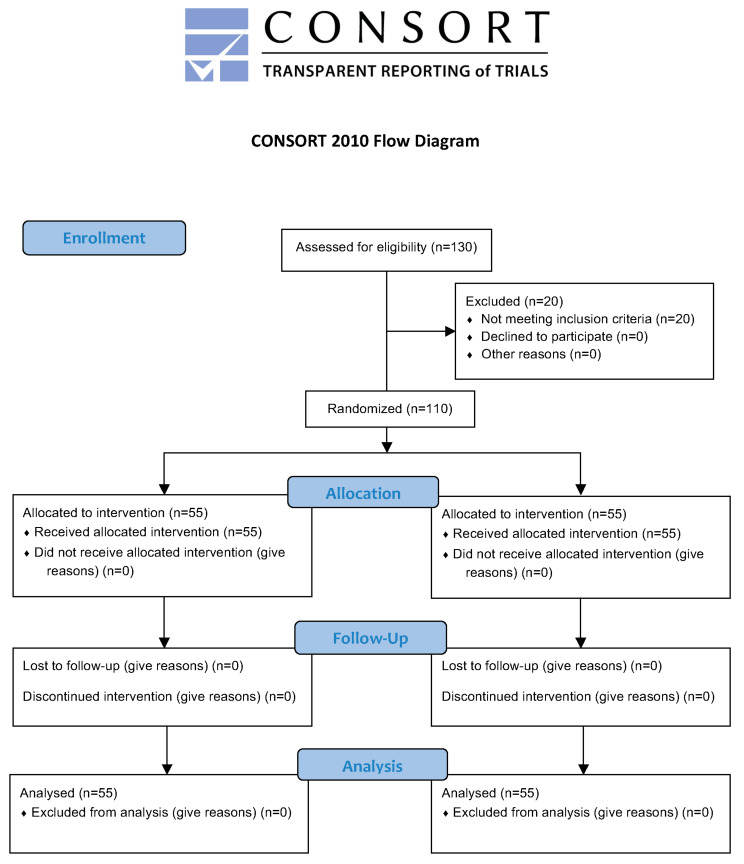
Consort flow diagram.

All the participants randomly assigned to the two study groups (SMB and RMB) completed the trial (Figure 4). Their distribution by sex was 35 women (63.6%) and 20 men (36.4%) in the RMB group (retromalleolar technique) and 34 women (61.8%) and 21 men (38.2%) in the SMB group (supramalleolar technique). Table 2 shows a comparison of the sample characteristics for the two study groups (RMB and SMB).

Table 3 shows comparisons of anesthesia times and levels of pain registered during the two anesthetic procedures. Student’s *t*-test found no statistically significant difference for any of the variables analyzed.

**Figure 5:**
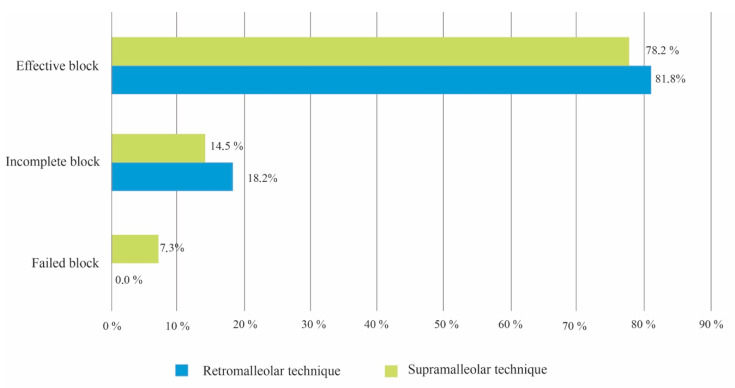
Comparison of the anesthetic results achieved with the two techniques.

Table 4 shows a comparison of the anesthetic results achieved with the two techniques. The Chi-squared test found no statistically significant difference in the success rates of the two procedures (*p* = 0.634) (Figure 5). Nor were significant differences between the two techniques found for vascular puncture percentage (Chi-squared test *p* = 0.507) or for peri-/intraneural puncture percentage (Chi-squared test *p* = 0.751).

**Figure 6:**
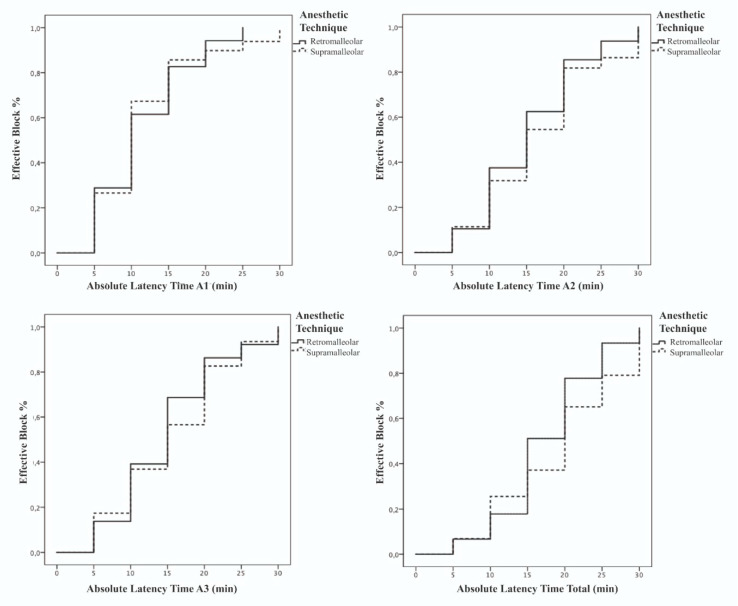
Comparison of the percentage of effective blocks based on the latency time for each of the areas and anesthetic techniques.

Figure 6 shows the percentage of effective blocks based on the latency time for each of the areas and anesthetic techniques, after carrying out the Kaplan–Meier analysis. The Mantel–Cox Log Rank (*p* = 0.114), Breslow (*p* = 0.271), and Tarone–Ware (*p* = 0.183) tests showed no statistically significant differences between the survival curves of the retromalleolar technique and the supramalleolar technique in any of the three areas into which the tibial nerve dermatome was divided.

## 4. Discussion

The initial hypothesis in the present trial was that blocking the tibial nerve at the supramalleolar level could achieve a higher rate of effective blocks, in that the nerve might be less likely to have forked into its terminal branches at that point. However, the trial results show not statistically significant differences between the two techniques.

The reason for the lack of statistical difference could be owing to the prior protocol that has been established for the two techniques. The injection site coordinates used in both techniques under study were based on a prior ultrasound study of the tibial nerve in a population of 100 subjects, which determined both the mean distance between the nerve and the osseous reference point and the depth of the nerve beneath the skin surface without directly touching the vascular and nerve bundle. In other words, ultrasound was used to determine, prior to performing the techniques, the injection site coordinates, not to perform echo-guided tibial nerve blocks. The purpose was to improve the efficiency of blocks administered by a conventional technique without instrumentation, that is, without the need for an ultrasound scanner. This would not only provide an economical advantage for the professional, but it would also increase the speed at which this anesthetic technique can be performed, as well as a reduction in the lacerations of the tissues caused by redirection of the needle.

The most prominent point of the medial malleolus was chosen as the reference point for the retromalleolar technique because it is an osseous prominence, which is easily recognized regardless of the anatomical characteristics of the subject’s ankle. The decision to block the nerve 4 cm proximally from the lower edge of the medial malleolus in the supramalleolar technique was based on the ultrasound image of the tibial nerve. A more proximal location of the nerve would have hindered correct ultrasound identification of the structure corresponding to the tibial nerve and, consequently, the definition of the target coordinates for the supramalleolar injection.

In the case of the retromalleolar anesthetic, no failed tibial nerve block was obtained. The same cannot be said of the supramalleolar technique, for which four failed blocks were recorded. These results highlight the greater difficulty in locating the tibial nerve at the supramalleolar level. A study by Larrabure et al. [19] also highlights the difficulty in locating the tibial nerve in a more proximal anatomical position. The tibial nerve dermatoma area that presented the greatest difficulty in achieving thermal and pain sensitivity blocking, with both the retromalleolar and the supramalleolar technique, was that of the medial plantar nerve. These results differ from those obtained by Hromádka et al. [10]. In their study, incomplete blocking of the tibial nerve was located in the lateral plantar nerve area.

As regards to anesthetic blocking of the calcaneus nerve dermatome (A1), the percentage of effective block achieved at the supramalleolar approach (89.1%) could corroborate a suggestion by some authors that the tibial nerve forks even more proximally than 4 cm above the lower edge of the medial malleolus [4,8,16,20].

Both of the tibial nerve blocking techniques used in the present trial were characterized by a single injection with no change in needle direction, a minimum effective volume of local anesthetic (3 mL), and an effective anesthetic verification time-out, in keeping with the onset of action time described for the anesthetic administered. The volume of anesthetic could explain why the retromalleolar and supramalleolar techniques under study obtained lower success rates than those reported by other authors based on high volume bloking [10,14,15,21] (Table 5). Specifically, with the minimum volume administered, the tibial nerve block success rate was 81.8% for the retromalleolar technique and 78.2% for the supramalleolar technique, although without significant differences between the two techniques. None of the subjects in whom the retromalleolar or supramalleolar technique achieved an effective tibial nerve block required intraoperative anesthetic reinforcement owing to insufficient analgesia. The mean blocking duration for both the retromalleolar technique and the supramalleolar technique guaranteed an adequate period of analgesia for any foot surgery procedure. Both study groups rated the pain perceived during the anesthetic process as low on the VAS.

Although the retromalleolar and supramalleolar techniques described in the present trial did not achieve 100% success rates, they did register a considerably higher number of effective blocks than those described for other conventional techniques [13,18], which are not based on a high volume tibial nerve block (22% and 66% of success rates reported, respectively). Indeed, the success rate with the retromalleolar technique was greater than that reported (72%) for the ultrasound-guided technique [13].

Future lines of research aim to investigate further the topographical anatomy of the tibial nerve and its relation to the posterior tibial artery in the ankle region. Determining if the foot type (rectus, planus, or cavus) influences the position of the tibial nerve could assist in adjusting the needle angle and puncture procedures and, if possible, further standardizing the needle insertion target points for tibial nerve blocking using a conventional technique without instrumentation. Authors also aim to establish an anesthetic technique for sural nerve block protocol to later compare the anesthetic efficiency obtained with that of the conventional technique described in the literature.

## 5. Conclusions

The two anesthetic techniques under study provided very similar clinical results. Contrary to the initial expectations, the supramalleolar approach did not achieve a higher effective blocking rate. The reason for the lack of statistical difference could be owing to the prior protocol that has been established for the two techniques. The injection coordinates (distance, needle depth, and needle angle) were obtained from prior ultrasound scans in order to locate the anatomical position of the tibial nerve at the retromalleolar and supramalleolar levels based on the sex and the height of the subjects. These scans provided the anatomical coordinates for optimum identification of the injection site for tibial nerve blocking.

Both anesthetic techniques were well accepted by the subjects and registered a considerably higher number of effective blocks than those that are not based on a high volume tibial nerve block. Both anatomic-landmark techniques were characterized by a single injection with no change in needle direction and a minimum effective volume of local anesthetic.

The greater ease in locating the tibial nerve in the retromalleolar approach could suggest that this is the technique of choice for tibial nerve blocking, especially in the case of professionals new to the field. Supramalleolar technique could be worth considering for those more experienced professionals and, particularly, in patients with restricted retromalleolar area access, owing, for example, to disorders or distortion of the ankle anatomy. In these cases, it would be recommended to perform echo-guided tibial nerve blocks to ascertain the anatomical position of the nerve with greater accuracy.

## Figures and Tables

**Table 1:** Injection site coordinates for both techniques obtained from prior ultrasound scans of the tibial nerve.

Injection Site Coordinates	Retromalleolar Technique				Supramalleolar Technique			
	Sex of Subject		Height of Subject (m)		Sex of Subject		Height of Subject (m)	
	Men	Women	<1.72	>1.72	Men	Women	<1.72	>1.72
Distance (cm) on a straiht line from the anatomical reference point	2.28–2.54	1.91–2.08	-	-	1.80–2.03	1.67–1.81	-	-
Vertical distance (cm) from the surface of the skin (needle depht)	0.9	0.9	-	-	1.30	1.10	-	-
Needle angle (°)	-	-	90	45			90	90

**Table 2:** Comparison of the sample characteristics for the two study groups (retromalleolar tibial nerve block (RMB) and supramalleolar tibial nerve block (SMB)). CI = confidence interval.

Sample Characteristics	Retromalleolar Technique (*n* = 55)		Supramalleolar Technique (*n* = 55)		Statistical Test
	Mean (Range)	95% CI	Mean (Range)	95% CI	Student’s *t*-Test
Age (years)	36.4 (18–75)	[31.4–41.3]	36.0 (19–75)	[31.3–40.8]	*p* = 0.920
Height (m)	1.66 (1.52–1.86)	[1.64–1.70]	1.68 (1.50–1.93)	[1.65–1.71]	*p* = 0.183
Weight (kg)	63.4 (43–98)	[59.7–67.1]	64.6 (45–87)	[61.7–67.5]	*p* = 0.616

**Table 3:** Comparisons of anesthesia times and levels of pain registered during the two anesthetic procedures. RLT = relative latency time, ALT = absolute latency time, VAS = visual analog scale.

Time & Pain Variables	Retromalleolar Technique (*n* = 55)		Supramalleolar Technique (*n* = 55)		Statistical Test
	Mean (Range)	95% CI	Mean (Range)	95% CI	Student’s *t*-Test
RLT (min)	3.26 (0.17–9.9)	[2.68–3.86]	4.1 (0.42–10.25)	[3.41–4.78]	*p* = 0.069
ALT A1 (min)	11.63 (5–25)	[9.98–13.28]	11.83 (5–30)	[9.88–13.8]	*p* = 0.874
ALT A2 (min)	15.51 (5–30)	[13.55–17.48]	16.66 (5–30)	[14.43–18.91]	*p* = 0.591
ALT A3 (min)	15 (5–30)	[13.03–16.96]	15.65 (5–30)	[13.45–19.51]	*p* = 0.657
ALT total (min)	17.93 (5–30)	[15.9–19.88]	19.28 (5–30)	[16.86–21.73]	*p* = 0.287
Effective anesthetic block duration (h)	2.5 (0.93–4.75)	[2.28–2.71]	2.51 (1.16–4.21)	[2.28–2.76]	*p* = 0.895
Pain level VAS score (0–10)	2.16 (0–8)	[1.64–2.68]	2.24 (0–7)	[1.69–2.79]	*p* = 0.847

RLT = relative latency time, ALT = absolute latency time, A1 = calcaneal nerve area, A2 = medial plantar nerve area, A3 = lateral plantar nerve area, min = minutes, h = hours, VAS = visual analog scale.

**Table 4:** Comparison of the anesthetic results achieved with the two techniques.

Anesthetic Results	Retromalleolar Technique (*n* = 55)	Supramalleolar Technique (*n* = 55)	Statistical Test
	%	%	Chi^2^
Vascular puncture	10.9	7.3	0.507
Peri/intraneural puncture	9.1	10.9	0.751
A_1_ block	94.5	89.1	0.297
A_2_ block	83.3	81.8	0.429
A_3_ block	92.7	83.6	0.140
Effective block	81.8	78.2	0.634
Incomplete block	18.2	14.5	0.606
Failed block	0	7.3	0.349

**Table 5:** Comparison of the success rates reported by other authors based on a high volume blocking with those obtained in the present study for the retromalleolar technique (R.T.) and supramalleolar technique (S.T.).

Author (Year of Publication)	Anesthetic Volume Administered	Success Rate Reported
Sarrafian et al. (1983) [15]	7–10 mL	94%
Colgrove (2001) [21]	7–10 mL	100%
Rudkin et al. (2005) [14]	10 mL	94.7%
Hromádka et al. (2010) [10]	15 mL	92.9%
Benimeli et al. (2020) (present study)	3 mL 3 mL	81.8% (R.T.) 78.2% (S.T.)

## Supplementary Materials

The following are available online at https://www.mdpi.com/1660-4601/17/11/3860/s1, Table S1: Consort Checklist.
